# Editorial: Novel sources of natural active compounds for skin protection and treatment of skin disorders

**DOI:** 10.3389/fphar.2024.1527849

**Published:** 2024-12-03

**Authors:** Katarzyna Gaweł-Bęben, Wirginia Kukula-Koch, Katerina Argyropoulou, Agnieszka Szopa

**Affiliations:** ^1^ Department of Cosmetology, University of Information Technology and Management, Rzeszów, Poland; ^2^ Department of Pharmacognosy with Medicinal Plants Garden, Medical University of Lublin, Lublin, Poland; ^3^ Division of Pharmacognosy and Natural Products Chemistry, Department of Pharmacy, National and Kapodistrian University of Athens, Athens, Greece; ^4^ Department of Medicinal Plant and Mushroom Biotechnology, Medical College, Jagiellonian University, Kraków, Poland

**Keywords:** natural products, cosmetics, antioxidants, anti-aging, skin-lightening, UV-protectant, anti-inflammatory, biotechnology

The use of natural raw materials in skin care and skin protection against the harmful effects of environmental factors has been the subject of intensive scientific research for many years. The use of modern extraction and preparation techniques of natural ingredients makes them more and more effective, safe and stable ingredients of cosmetics and dermatological products. The aim of this Research Topic was to present selected modern methods of obtaining natural raw materials and assessing their multi-directional, skin-protecting effects and their potential application in the treatment of selected skin disorders. An example of such study is the work presented by Huang et al., showing the potential application of *Chrysanthemi indic*i flower extract in cosmetics for sensitive skin. In this study the skin calming potential of *Ch. indic*i flower extract was supported by the significant potential of DPPH and ABTS radicals neutralization, and promising anti-inflammatory activity by inhibiting cytokines of IL-1β, IL-6, PEG2, TNF-α, IFN-γ and NO in LPS induced RAW264.7 cells in a dose-dependent manner. Using the UF-LC/MS method the authors detected linarin and chlorogenic acid isomers as main bioactive components responsible for the anti-inflammatory and sensitive skin improvement activities.

In another study presented within this Research Topic Albouy et al. indicated that bio-fermented *Aframomum angustifolium* extract treatments have pleiotropic beneficial effects on skin equivalents and that the bio-fermentation provides new biological activities to this plant. The bio-fermented *A. angustifolium* extract contained specific organic acids such as lactic, gluconic, succinic acid and polyphenols. Keratinocyte stem cells -depleted skin equivalents that were treated with bio-fermented *A. angustifolium* extract exhibited higher specular reflection, indicating better hydration of the stratum corneum, higher mitotic activity in the epidermis basal layer, improved dermal-epidermal connectivity, and increased rigidity of the dermal-epidermal junction compared to non-treated keratinocyte stem cells -depleted equivalents. Bio-fermented *A. angustifolium* extract treatments also resulted in changes at the dermis level, with an increase in total collagen and a decrease in global laxity, suggesting that this extract could help maintain young-looking skin.

Liu et al. proved that polypeptide P-8-R from *Eleutherococcus sessiliflorus* has a strong antioxidant effect. It not only prevents oxidative stress damage and apoptosis in skin cells but also inhibits skin collagen loss, thus preventing skin aging. This study contributes to a deeper understanding of the effective material basis of *E. sessiliflorus*, and suggests that P-8-R could be a potential antioxidant drug or an active ingredient of skin care cosmetics in the future.

In the next research article Tietel et al. provided significant evidence of the anti-inflammatory and regenerative potential of Jojoba (*Simmondsia chinensis* L.) wax following topical application. The treatment of human *ex-vivo* skin explants with Jojoba wax significantly reduced LPS-induced secretion of proinflammatory cytokines IL-6, IL-8, and TNF-α, increasing the release of anti-inflammatory TGF-β1. Jojoba wax treatment also increased the expression of collagen III and hyaluronic acid.

In their studies Arshad et al. investigated the rejuvenating potential of a cream enriched with 4% *Isatis argentea* extract. Due to a high content of polyphenols proved by the HPLC-PDA and spectrophotometric assays, the results confirmed its marked whitening potential, and promising sun protection effects. In the tests on human trial subjects a substantial drop in melanin was noted within 12 weeks, whereas both skin moisture and elasticity indices were found to be elevated.

Al-Ghanayem presented an interesting study on anti-acne, anti-inflammatory and antioxidant potential of the methanolic extract from *Teucrium oliverianum* that was determined, among others, in HaCaT cells infected with *Cutibacterium acnes*. The major metabolites with esculin, L-carnitine and gamma-linoleic acid attenuated the cytotoxicity of the bacterium at 676.2 μg/mL, while the anti-inflammatory potential was expressed as decreased levels of IL-1β, INF-γ, COX-2 and TNF-α.

This Research Topic also includes two review articles that shed new light on the cosmetic and medicinal uses of two examples of well-known ingredients of cosmetics and wound healing preparations. Oargă Porumb et al. summarized the current knowledge on the application of rosehip oil and extracts in formulations of modern cosmetics. Significant anti-aging, skin-lightening and anti-inflammatory properties of rosehip products are known for a long time, but using modern technologies for extraction and efficient delivery of rosehip products (e.g., using nano-carriers) into the deeper epidermal layers might significantly increase the benefits coming from the Research Topic use of these valuable natural ingredients. The application of gelatin and gelatin-based biomaterials in wound healing has been summarized in a review article by (Cao et al.). Due to the wide availability, relatively low cost, good biocompatibility and degradability gelatin is a valuable component of modern wound dressing. Gelatin-based wound dressings help to maintain tissue homeostasis, promote vascular and epithelial regeneration, and might be loaded with anti-inflammatory drugs to reduce the inflammation at the wound site. Major challenges of the use of gelatin-based biomaterials in wound healing are their poor mechanistic properties and low antibacterial activity. The review article presents several possibilities to overcome these drawbacks and expand the application of gelatin-based biomaterials in wound healing and repair.

We believe that this Research Topic presents the wide possibilities of using new sources of natural ingredients in skin care, and therapies of selected skin problems. It also presents the need to conduct further research on improving the methods of obtaining natural raw materials, and assessing the cosmetic value of ingredients of natural origin.

